# NTCE-KD: Non-Target-Class-Enhanced Knowledge Distillation

**DOI:** 10.3390/s24113617

**Published:** 2024-06-03

**Authors:** Chuan Li, Xiao Teng, Yan Ding, Long Lan

**Affiliations:** College of Computer Science and Technology, National University of Defense Technology, Changsha 410073, China; lichuan18@nudt.edu.cn (C.L.); tengxiao14@nudt.edu.cn (X.T.); yanding@nudt.edu.cn (Y.D.)

**Keywords:** knowledge distillation, adaptive distillation, data augmentation, model compression, image classification, deep learning

## Abstract

Most logit-based knowledge distillation methods transfer soft labels from the teacher model to the student model via Kullback–Leibler divergence based on softmax, an exponential normalization function. However, this exponential nature of softmax tends to prioritize the largest class (target class) while neglecting smaller ones (non-target classes), leading to an oversight of the non-target classes’s significance. To address this issue, we propose Non-Target-Class-Enhanced Knowledge Distillation (NTCE-KD) to amplify the role of non-target classes both in terms of magnitude and diversity. Specifically, we present a magnitude-enhanced Kullback–Leibler (MKL) divergence multi-shrinking the target class to enhance the impact of non-target classes in terms of magnitude. Additionally, to enrich the diversity of non-target classes, we introduce a diversity-based data augmentation strategy (DDA), further enhancing overall performance. Extensive experimental results on the CIFAR-100 and ImageNet-1k datasets demonstrate that non-target classes are of great significance and that our method achieves state-of-the-art performance across a wide range of teacher–student pairs.

## 1. Introduction

With the rapid advancements in deep learning, neural networks have undergone significant development, achieving remarkable breakthroughs in diverse domains including image classification [1,2,3], object detection and tracking [4,5,6,7], and semantic segmentation [8,9]. However, despite their impressive performance, these models typically require substantial computational and storage resources, posing challenges for practical deployment on devices like intelligent sensors.

Given the typical resource constraints of intelligent sensors, such as limited memory and computational capabilities, knowledge distillation (KD) emerges as a particularly promising solution [10]. KD enables the transfer of intricate knowledge from heavyweight teacher models to lightweight student models, allowing the latter to achieve comparable performance while significantly reducing resource requirements. This approach is particularly relevant in the context of intelligent sensors, where efficient utilization of resources is crucial for effective real-world deployment.

KD is primarily categorized into two branches: logit-based distillation and feature-based distillation. Logit-based methods transfer knowledge by minimizing the Kullback–Leibler (KL) divergence. Conversely, feature-based methods leverage knowledge from deep intermediate layers for superior performance at the cost of computational demands. However, logits with higher semantic information are supposed to provide more “dark knowledge” and logit-based methods are supposed to achieve better or comparable performance as feature-based methods, theoretically. Therefore, we believe that the knowledge within the logits (i.e., soft labels) of the teacher model has not been fully exploited.

**Figure 1:**
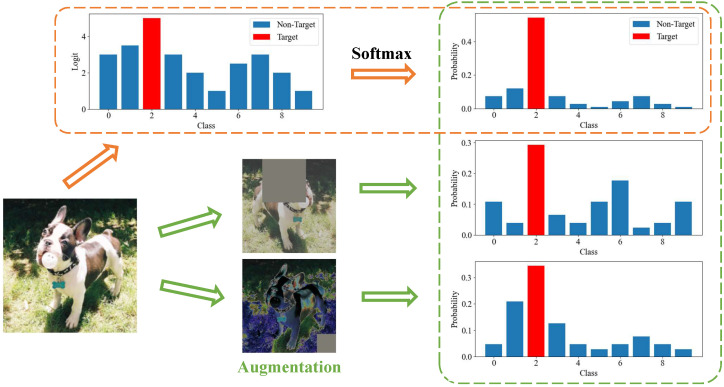
Motivation. Orange dashed box: the exponential nature of softmax results in overlooking the effect of non-target classes. Green dashed box: viewing the same sample from different perspectives provides diverse insights into category relevance.

Soft labels of the teacher model encompass the target class logit along with the non-target class logits. The target class involves knowledge of the sample’s true category, whereas the non-target class contains rich knowledge of category relevance. The soft labels are obtained by applying a softmax function to logits. However, the softmax function commonly utilized in most existing logit-based methods tends to disproportionately accentuate the largest class due to its exponential nature, thereby overlooking the informative guidance within the non-target class, as shown in the orange dashed box within Figure 1.

Moreover, considering that the same sample may appear in different categories from different perspectives (green dashed box in Figure 1) thereby provides diverse knowledge of category relevance. Transferring knowledge of samples from a single perspective fails to fully exploit the latent inter-class correlation within the sample.

To address these issues, we propose a flexible and efficient logit-based distillation method dubbed Non-Target-Class-Enhanced Knowledge Distillation (NTCE-KD) to enhance the role of the non-target class in terms of both magnitude and diversity. Firstly, we introduce a magnitude-enhanced KL (MKL) divergence, in which the teacher logits’ target class is multi-shrunk before applying softmax, yielding more informative soft labels rich in non-target class knowledge. To ensure the convergence of the model, identical compensatory logit shrinkage is applied to the student’s target class. Moreover, to explore the diverse categorical relevance knowledge within the non-target class, we present a diversity-based data augmentation strategy (DDA) to obtain samples’ various views.

Overall, our contributions can be summarized as follows:

- We reveal the effect of non-target classes and present an improved KL divergence, named MKL, achieved by applying multi-shrinkages to the target class logits of both the teacher and student, thus amplifying the role of non-target classes in terms of magnitude.
- We demonstrate that different views of an identical sample yield varying levels of similarity knowledge among categories. To enhance the diversity of non-target classes, we introduce a data augmentation strategy named DDA.
- We propose NTCE-KD, a novel approach that enhances the significance of non-target classes in terms of both magnitude and diversity. We conduct extensive experiments on CIFAR-100 [11] and ImageNet-1k [12] across various teacher–student pairs, demonstrating our model’s significant superiority.

## 2. Related Work

### Knowledge Distillation

Knowledge distillation, introduced by Hinton et al. [10], stands as an efficient model compression technique. Its core objective is to transfer the learned knowledge from a teacher model to a student model. Within the realm of knowledge distillation, two primary methods have gained significant attention: logit-based distillation and feature-based distillation.

Logit-based distillation methods [10] primarily focus on aligning the output logits of the teacher and student models. This approach offers a straightforward and practical solution for knowledge transfer. Conversely, feature-based distillation methods [13,14,15,16] emphasize the alignment of intermediate features extracted from the hidden layers of the teacher model. While these feature-based methods often demonstrate impressive performance, they tend to introduce significant computational overhead. This can render them impractical in scenarios where accessing intermediate features poses challenges.

Current logit-based distillation methods often require the student model to mimic the softmax-standardized soft labels of the teacher model, potentially overlooking knowledge within the non-target class. To improve knowledge transfer, certain adaptive logit-based distillation methods have been proposed, which may inadvertently increase the influence of the non-target class to some extent. ATS [17] employs a lower temperature for the target class compared to the non-target class. Several other approaches [18,19,20] adjust specific temperatures to globally scale the soft labels. However, these temperature-based methods are restricted to globally scaling soft labels, limiting their ability to flexibly explore knowledge within both target and non-target classes. DKD [21] aims to enhance the teacher’s soft labels by decoupling the target and non-target classes from KL divergence into a fixed proportion, but it lacks sample-wise enhancement for the non-target class. Moreover, the diversity of the non-target distribution remains underexplored. Recognizing these limitations, our NTCE-KD explicitly enhances the magnitude and diversity of the non-target, thereby amplifying the role of the non-target class.

## 3. Materials and Methods

### 3.1. Preliminaries

Consider a dataset of image classification containing *N* samples ${\left\{x_{n},y_{n}\right\}}_{n=1}^{N}$, where $x_{n}\in R^{H\times W}$ is the *n*-th sample and $y_{n}\in \left[1,K\right]$ is its corresponding label. The notations *H*, *W* are the height and width of the image, and *K* is the total class number of the dataset. Consider the teacher model $f_{T}$, the student model $f_{S}$, the logits of teacher $z_{n}=f_{S}\left(x_{n}\right)$, and the logits of student $v_{n}=f_{T}\left(x_{n}\right)$, where $z_{n}$ and $v_{n}\in R^{1\times K}$.

It is widely accepted that the predicted probability vectors $p(z_{n},\tau )$ and $p(v_{n},\tau )$ are standardized by softmax, and the *k*-th classes of these predicted probability vectors $p{\left(z_{n},\tau \right)}^{\left(k\right)}$ and $p{\left(v_{n},\tau \right)}^{\left(k\right)}$ are calculated as follows:

(1) $$\begin{array}{cc} p{\left(z_{n},\tau \right)}^{\left(k\right)}= & \frac{\exp(z_{n}^{\left(k\right)}/\tau )}{\sum_{m=1}^{K}\exp(z_{n}^{\left(m\right)}/\tau )}, \end{array}$$

(2) $$\begin{array}{cc} p{\left(v_{n},\tau \right)}^{\left(k\right)}= & \frac{\exp(v_{n}^{\left(k\right)}/\tau )}{\sum_{m=1}^{K}\exp(v_{n}^{\left(m\right)}/\tau )}, \end{array}$$

where τ is the temperature to soften the probability. Notably, $k=y_{n}$ presents the target class of the probability vectors and $k\neq y_{n}$ presents the non-target class.

Knowledge distillation aims to align the predicted probability vectors $p{\left(z_{n},\tau \right)}^{\left(k\right)}$ of the student to the soft labels $p{\left(v_{n},\tau \right)}^{\left(k\right)}$ for each class via KL divergence.

(3) $$\begin{array}{cc} L_{KL} & =\sum_{n=1}^{N}KL(p\left(v_{n},\tau \right)||p\left(z_{n},\tau \right))\\ & =\sum_{n=1}^{N}\sum_{k=1}^{K}p{\left(v_{n},\tau \right)}^{\left(k\right)}\log\frac{p{\left(v_{n},\tau \right)}^{\left(k\right)}}{p{\left(z_{n},\tau \right)}^{\left(k\right)}}, \end{array}$$

where $L_{KL}$ is the knowledge distillation loss. The temperature τ is set greater than 1 to produce softer probability vectors for conveying more information.

In addition to the soft labels, it is generally beneficial to train the student together with ground-truth labels via the cross-entropy loss $L_{CE}$.

(4) $$\begin{array}{cc} L_{CE}= & -\sum_{n=1}^{N}p{\left(z_{n}\right)}^{\left(y_{n}\right)}\log p{\left(z_{n},\tau =1\right)}^{\left(y_{n}\right)}, \end{array}$$

where the temperature τ is set as 1.

The overall optimization objective involves both the knowledge distillation loss $L_{KD}$ and the cross-entropy loss $L_{CE}$.

(5) $$\begin{array}{cc} L_{total}= & \alpha L_{CE}+\beta L_{KL} \end{array}$$

where α and β are weights for balancing the losses.

### 3.2. Magnitude Enhancement

**Figure 2:**
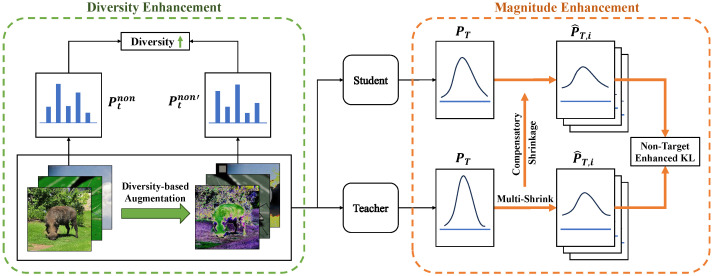
The framework of our proposed NTCE-KD with magnitude and diversity enhancements in orange and green dashed boxes, respectively. Magnitude enhancement: multi-shrink the target class of the teacher’s logits and apply the same shrinkage to the target class of the student’s logits. Diversity enhancement: seek data augmentations to maximize the diversity of samples’ non-target classes.

In Section 3.2.1 and Section 3.2.2, we show the effect of the non-target class on knowledge distillation from a mathematical perspective and the magnitude side-off drawbacks of the original KL divergence. In Section 3.2.3, we propose a magnitude-enhanced KL divergence by shrinking the target class as shown in Figure 2.

#### 3.2.1. Effect of Non-Target

In the classification tasks, the non-target classes refer to those categories that are not selected as the predicted class in a given sample. In this section, we analyze the effect of the non-target class from the perspectives of reinterpretation and gradient.

Reinterpretation of KL. KL divergence in Equation (3) can be interpreted as the cumulative weighted difference across all classes:

(6) $$\begin{array}{cc} L_{KL}= & \sum_{n=1}^{N}\sum_{k=1}^{K}p{\left(v_{n},\tau \right)}^{\left(k\right)}\log\frac{p{\left(v_{n},\tau \right)}^{\left(k\right)}}{p{\left(z_{n},\tau \right)}^{\left(k\right)}}\\ = & \sum_{n=1}^{N}\sum_{k=1}^{K}p{\left(v_{n},\tau \right)}^{\left(k\right)}\left(\log p{\left(v_{n},\tau \right)}^{\left(k\right)}-\log p{\left(z_{n},\tau \right)}^{\left(k\right)}\right), \end{array}$$

where $p{\left(v_{n},\tau \right)}^{\left(k\right)}$ is the *k*-th weight for difference and $\log p{\left(v_{n},\tau \right)}^{\left(k\right)}-\log p{\left(z_{n},\tau \right)}^{\left(k\right)}$ is the difference in the *k*-th class.

Enhanced model generalization. Equation (6) aims to align the logarithmic probability of both target and non-target classes between teacher and student. Thus, the model learns to discriminate not only between the correct and incorrect classes but also among various incorrect classes, enhancing the model’s generalization ability when faced with unseen or difficult data.

Derivation of gradient. To further analyze the optimization of the student when aligned with the teacher, we calculate the gradient of $L_{KL}$ with respect to $z^{\left(k\right)}$, omitting τ and *n* for brevity.

Taking the partial derivative with respect to $p{\left(z\right)}^{\left(k\right)}$ gives

(7) $$\begin{array}{cc} \frac{\partial L_{KL}}{\partial p{\left(z\right)}^{\left(k\right)}}= & -\frac{p{\left(v\right)}^{\left(k\right)}}{p{\left(z\right)}^{\left(k\right)}}. \end{array}$$

Taking the partial derivative of $p{\left(z\right)}^{\left(k\right)}$ with respect to $z^{\left(i\right)}$ for i∈[1,C], when i=k, we obtain

(8) $$\begin{array}{cc} \frac{\partial p{\left(z\right)}^{\left(k\right)}}{\partial z^{\left(k\right)}}= & \frac{\partial (\frac{\exp\;z^{\left(k\right)}}{\sum_{j=1}^{K}\exp\;z^{\left(j\right)}})}{\partial z^{\left(k\right)}}\\ = & \frac{\exp\;z^{\left(k\right)}\sum_{j=1}^{K}\exp\;z^{\left(j\right)}-{\left(\exp\;z^{\left(k\right)}\right)}^{2}}{{\left(\sum_{j=1}^{K}\exp\;z^{\left(j\right)}\right)}^{2}}\\ = & \left(\frac{\exp\;z^{\left(k\right)}}{\sum_{j=1}^{K}\exp\;z^{\left(j\right)}}\right)\left(1-\frac{\exp\;z^{\left(k\right)}}{\sum_{j=1}^{K}\exp\;z^{\left(j\right)}}\right)\\ = & p{\left(z\right)}^{\left(k\right)}\left(1-p{\left(z\right)}^{\left(k\right)}\right). \end{array}$$

When i≠k, we obtain

(9) $$\begin{array}{cc} \frac{\partial p{\left(z\right)}^{\left(k\right)}}{\partial z^{\left(i\right)}}= & \frac{\partial (\frac{\exp\;z^{\left(k\right)}}{\sum_{j=1}^{K}\exp\;z^{\left(j\right)}})}{\partial z^{\left(i\right)}}\\ = & \frac{-\exp\;z^{\left(k\right)}\exp\;z^{\left(i\right)}}{{\left(\sum_{j=1}^{K}\exp\;z^{\left(j\right)}\right)}^{2}}\\ = & -p{\left(z\right)}^{\left(k\right)}p{\left(z\right)}^{\left(i\right)}. \end{array}$$

Based on Equations (7)–(9) and the chain rule, the partial derivative of $L_{KL}$ with respect to *z* can be derived as follows:

(10) $$\begin{array}{cc} \frac{\partial L_{KL}}{\partial z^{\left(k\right)}}= & \sum_{j=1}^{K}\left(\frac{\partial L_{KL}}{\partial p{\left(z\right)}^{\left(j\right)}}\frac{\partial p{\left(z\right)}^{\left(j\right)}}{\partial z^{\left(k\right)}}\right)\\ = & \sum_{j=1,j\neq k}^{K}\left(\frac{\partial L_{KL}}{\partial p{\left(z\right)}^{\left(j\right)}}\frac{\partial p{\left(z\right)}^{\left(j\right)}}{\partial z^{\left(k\right)}}\right)+\left(\frac{\partial L_{KL}}{\partial p{\left(z\right)}^{\left(k\right)}}\frac{\partial p{\left(z\right)}^{\left(k\right)}}{\partial z^{\left(k\right)}}\right)\\ = & \sum_{j=1,j\neq k}^{K}\left(-\frac{p{\left(v\right)}^{\left(j\right)}}{p{\left(z\right)}^{\left(j\right)}}*-p{\left(z\right)}^{\left(j\right)}p{\left(z\right)}^{\left(k\right)}\right)+\left(-\frac{p{\left(v\right)}^{\left(k\right)}}{p{\left(z\right)}^{\left(k\right)}}*p{\left(z\right)}^{\left(k\right)}\left(1-p{\left(z\right)}^{\left(k\right)}\right)\right)\\ = & p{\left(z\right)}^{\left(k\right)}-p{\left(v\right)}^{\left(k\right)}. \end{array}$$

Achievable optimization objective. The optimization objective of cross-entropy loss is to maximize the target class. The ideal output for the student is in the one-hot format, which is challenging for a model to achieve. However, the knowledge distillation loss compels the student to produce a probability identical to that of the teacher, as shown in Equation (10). With the temperature τ, the teacher’s output is more reasonable and achievable for the student model. Thus, the non-target classes offer an achievable optimization objective for the student.

#### 3.2.2. Drawbacks of KL

Based on the advantages of the non-target class listed above, we analyze the shortcomings of the original KL divergence.

Inadequate non-target class optimization. From Equation (10), the optimizing magnitude for *k*-th class ${|p\left(z\right)}^{\left(k\right)}-p{\left(v\right)}^{\left(k\right)}|$ during the distillation is the absolute difference between the probabilities of teacher and student. However, the target class generally receives a much higher probability compared to non-target classes. This discrepancy leads to stronger gradients and, consequently, more focused optimization on the target class at the expense of the non-target classes.

**Figure 3:**
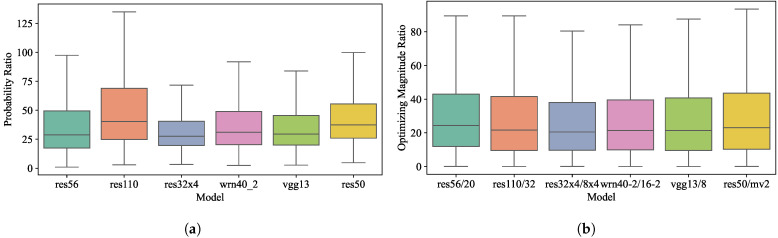
Statistical support for inadequate non-target class optimization. (**a**) r1: ratio of the probability of the target class to that of the non-target classes; (**b**) r2: ratio of the optimizing magnitude of the target class to that of the non-target classes.

Statistical support. To verify the inadequate optimization of the non-target class, we define two variables.

(11) $$\begin{array}{cc} r1= & \frac{p{\left(v\right)}^{\left(y_{n}\right)}}{\frac{1}{K-1}\sum_{k=1,k\neq y_{n}}^{K}p{\left(v\right)}^{\left(k\right)}}, \end{array}$$

(12) $$\begin{array}{cc} r2= & \frac{{|p\left(v\right)}^{\left(y_{n}\right)}-p{\left(z\right)}^{\left(y_{n}\right)}|}{\frac{1}{K-1}\sum_{k=1,k\neq y_{n}}^{K}\left|p{\left(v\right)}^{\left(k\right)}-p{\left(z\right)}^{\left(k\right)}\right|}, \end{array}$$

where r1 represents the ratio of the probability of the target class to the average probability of the non-target classes, and r2 measures the ratio of the optimizing magnitude of the target class to the average optimizing magnitude of the non-target classes. As indicated in Figure 3, statistical data from various teacher–student pairs consistently indicate that probabilities for target classes exceed those for non-target classes. Moreover, the target class exhibits a greater optimizing magnitude compared to the non-target classes. These findings validate our analysis that models tend to prioritize target class optimization, resulting in a disproportionate focus during the training phase.

#### 3.2.3. Magnitude-Enhanced KL

To explicitly enhance the role of the non-target classes in optimization, we seek to introduce a non-target-class-enhanced KL divergence to increase the magnitude of probabilities of non-target classes.

Target class multi-shrinkage. Taking into account the suppressive effect of an excessively large target class on the non-target classes, we propose to shrink the target class in the logits and apply softmax to normalize them, increasing the non-target class logits globally.

(13) $$\begin{array}{cc} {\tilde{v}}_{n}^{\left(y_{n}\right)}= & v_{n}^{\left(y_{n}\right)}-S_{n}, \end{array}$$

where $S_{n}$ is the shrinkage for the target class of the *n*-th sample. To strike a balance between emphasizing non-target classes and maintaining the discriminative power of the model for accurate classification, we introduce the base shrinkage $S_{n0}$ as the difference between the target class and the maximum non-target class.

(14) $$\begin{array}{cc} S_{n0}= & v_{n}^{\left(y_{n}\right)}-\max_{k\in \left[1,K\right],k\neq y_{n}}v_{n}^{\left(k\right)}. \end{array}$$

To further enrich the information in the soft labels, we utilize a shrinkage coefficient $\lambda_{m}$ to derive the *m*-th shrinkage $\lambda_{m}*S_{n0}$, where $\lambda \in D_{\lambda }=\left\{\lambda_{m}|1\leq m\leq M\right\}$ and *M* is the total number of the shrinkage coefficient. With the multi-shrinkages, the scaled target class is

(15) $$\begin{array}{cc} {\tilde{v}}_{n,m}^{\left(y_{n}\right)}= & v_{n}^{\left(y_{n}\right)}-\lambda_{m}*S_{n0}. \end{array}$$

And the *m*-th multi-shrunk probability of teacher is

(16) $$\begin{array}{c} p{\left({\tilde{v}}_{n,m}\right)}^{\left(k\right)}=\left\{\begin{array}{cc} \frac{\exp\left({\tilde{v}}_{n,m}^{\left(y_{n}\right)}\right)}{\sum_{m=1,m\neq y_{n}}^{K}\exp(v_{n}^{\left(m\right)})+\exp\left({\tilde{v}}_{n,m}^{\left(y_{n}\right)}\right)}, & \mathrm{if}\;k=y_{n}\\ \frac{\exp\left(v_{n}^{\left(k\right)}\right)}{\sum_{m=1,m\neq y_{n}}^{K}\exp(v_{n}^{\left(m\right)})+\exp\left({\tilde{v}}_{n,m}^{\left(y_{n}\right)}\right)}. & \mathrm{otherwise} \end{array}\right. \end{array}$$

Compensatory shrinkage for convergence. To ensure convergence, the same multi-shrinkages are applied to the target class of student’s logits.

(17) $$\begin{array}{cc} {\tilde{z}}_{n,m}^{\left(y_{n}\right)}= & z_{n}^{\left(y_{n}\right)}-\lambda_{m}*S. \end{array}$$

And the *i*-th multi-shrunk probability of the student is

(18) $$\begin{array}{c} p{\left({\tilde{z}}_{n,m}\right)}^{\left(k\right)}=\left\{\begin{array}{cc} \frac{\exp\left({\tilde{z}}_{n,m}^{\left(y_{n}\right)}\right)}{\sum_{m=1,m\neq y_{n}}^{K}\exp(z_{n}^{\left(m\right)})+\exp\left({\tilde{z}}_{n,m}^{\left(y_{n}\right)}\right)}, & \mathrm{if}\;k=y_{n}\\ \frac{\exp\left(z_{n}^{\left(k\right)}\right)}{\sum_{m=1,m\neq y_{n}}^{K}\exp(z_{n}^{\left(m\right)})+\exp\left({\tilde{z}}_{n,m}^{\left(y_{n}\right)}\right)}. & \mathrm{otherwise} \end{array}\right. \end{array}$$

The magnitude-enhanced KL divergence is derived as follows:

(19) $$\begin{array}{cc} L_{MKL}= & \frac{1}{M}\sum_{n=1}^{N}\sum_{m=1}^{M}KL(p\left({\tilde{v}}_{n,m},\tau \right)||p\left({\tilde{z}}_{n,m},\tau \right)). \end{array}$$

The improved KL divergence we devised possesses three key characteristics:

- Prominent role of the target class. For any given sample $x_{n}$, the magnitude of optimization for the student model on the target class is always the greatest, i.e., $\frac{\partial L_{KL}}{\partial z^{\left(y_{n}\right)}}>\frac{\partial L_{KL}}{\partial z^{\left(k\right)}}$ holds for all $k\neq y_{n}$. This ensures that sufficient attention is given to the target class in the new KL divergence formulation, thereby enabling more accurate predictions.
- Isotonicity among the non-target classes. Given the indices $t_{1},...,t_{k-1}$ that sort the original probabilities of the teacher, such that $p{\left(v_{n,m}\right)}^{\left(t_{1}\right)}<...<p{\left(v_{n,m}\right)}^{\left(t_{k}\right)}$ for $k\neq y_{n}$, it is observed that the ratio $\frac{p{\left(v_{n,m}\right)}^{k}}{p{\left({\tilde{v}}_{n,m}\right)}^{k}}$ remains constant at $\frac{1}{c}$, where *c* is a constant. This implies that the ordering of non-target probabilities $p{\left({\tilde{v}}_{n,m}\right)}^{\left(t_{1}\right)}<...<p{\left({\tilde{v}}_{n,m}\right)}^{\left(t_{k}\right)}$ is preserved for the teacher’s model. Similarly, it can be proven that the ordering of non-target probabilities is also preserved in the student’s model, preserving the underlying structure of class relationships, which is critical for meaningful learning.
- Convergence property. Assuming identical logits between the teacher and student models, i.e., $v_{n}^{\left(k\right)}=z_{n}^{\left(k\right)}$ for all k∈[1,K], it is evident that $L_{MKL}$ remains constant at zero, thus ensuring stability and rationality in the training process.

### 3.3. Diversity Enhancement

In order to further enhance the influence of the non-target class, drawing inspiration from multi-view learning, we increase the diversity of the non-target class in soft labels by employing specialized data augmentation techniques to generate diverse variations in samples.

#### Diversity-Based Data Augmentation

Previous studies have firmly established that the key benefit of implementing data augmentation policies lies in broadening the diversity of examples [22,23,24,25].

We seek an augmentation strategy that can enhance the diversity of the non-target classes based on *T* data transformations that are commonly used. To exert more precise control over the augmentation strategy, we introduce the two hyper-parameters *a* and *b* for the occurrences and intensity of transformations, respectively, following [22].

Given the occurrences of transformations *a*, each transformation will be selected with a probability 1/T, and there are $T^{a}$ potential augmentation strategies available. Additionally, *b* is set to adjust the transformation strength due to its significant impact on the diversity of the augmentation strategy. Specifically, the intensity of each transformation ranges from 0 to 10, with 10 representing the maximum transformation intensity, following [22].

Once *a* and *b* are specified, the data augmentation strategy can ultimately be represented as $\hat{X}=DataAug\left(a,b\right)$. The final dataset is $\hat{X}\cup X$, where X is the original dataset.

We propose a gradient-free search method to find the data augmentation strategy with the maximum diversity of non-target classes from all potential data augmentation distributions. The candidate values for *a* and *b* are set to $a=\{a_{1},a_{2},\ldots ,a_{n_{a}}\}$ and $b=\{b_{1},b_{2},\ldots ,b_{n_{b}}\}$, where $n_{a}$ and $n_{b}$ are the lengths of the two candidate sets. The objective of the search is listed as follows:

(20) $$\begin{array}{c} \underset{a\in a,b\in b}{\arg\min}\sum_{n=1}^{N}\sum_{k=1,k\neq y_{n}}^{K}\mathrm{Sim}\left(p{\left(f_{T}\left({\hat{X}}_{n}\right)\right)}^{\left(k\right)},p{\left(f_{T}\left(X_{n}\right)\right)}^{\left(k\right)}\right), \end{array}$$

where $X_{n}$ and ${\hat{X}}_{n}$ are the original version and the augmented version of the *n*-th sample, respectively, and Sim is the cosine similarity of the non-target class before and after the augmentation.

It is worth mentioning that the search process is conducted with the teacher model before distillation, effectively minimizing computational overhead. Moreover, despite the universal suitability of data augmentation for most distillation methods, we demonstrate in Section 4.5 that our proposed data augmentation approach can indeed enhance the diversity of non-target classes in samples to some extent, thereby improving the distillation performance.

## 4. Results

### 4.1. Datasets and Settings

Datasets. We evaluate our approach on CIFAR-100 [11] and ImageNet-1k [12]. CIFAR-100 [11] contains 60,000 images for 100 classes, with 50,000 images each in the training set and in the validation set. ImageNet-1k [12] is a large-scale dataset for image classification consisting of 1.2 million training and 50,000 validation images for 1000 classes.

Settings. We conduct our experiments on various teacher–student pairs of the same and different architectures, as shown in Table 1. Various neural network structures are utilized in the experiments, including ResNet [1], WRN [26], VGG [27], ShuffleNet-V1 [28]/V2 [2], and MobileNetV1 [29]/v2 [30].

Baselines. We compare our methods with various SOTA methods, including logit-based methods, such as KD [10], DKD [21], CTKD [19], DOT [31], and LS [32], and feature-based methods, such as FitNet [13], AT [16], RKD [33], OFD [34], CRD [35], ReviewKD [15], and CAT [36].

Implementation details. For a fair comparison, we conduct our experiments on standard teacher–student pairs following [15,21,35]. For CIFAR-100, we set the batch size to 64, the epoch to 240, the weight decay to $5\times 10^{-4}$, and the temperature parameter to 4. We set the initial learning rate at 0.05 for VGG and ResNet and 0.01 for ShuffleNet and MoblieNet. The learning rate is divided by 10 at 150, 180, and 210 epochs. For ImageNet, we set the batch size to 512, the epoch to 100, the weight decay to $1\times 10^{-4}$, and the temperature parameter to 1. The base learning rate is set to 0.2 and divided by 10 for every 30 epochs. For both datasets, we adopt an SGD optimizer with a momentum of 0.9. Our method is implemented in Pytorch. We train the model on a single GPU for CIFAR-100 and on four GPUs for ImageNet. The loss weights α and β are determined as 1.0 and 8.0, respectively, through grid search, and the shrinkage coefficients $D_{\lambda }$ is set to {1,0.5,0}. The determination of these hyper-parameters is discussed in detail in Section 4.6.

The data augmentation strategy consists of data transforms as follows: identity, autoContrast, equalize, rotate, solarize, color, posterize, contrast, brightness, sharpness, shear-x, shear-y, translate-x, translate-y. The conventional data augmentation strategy (DA) is DataAug(a,b), with randomly selected *a* and *b* in a and b.

### 4.2. Main Results

CIFAR-100. We compare our experimental results of CIFAR-100 with other KD methods on teacher–student pairs in the same architecture (Table 1) and different architectures (Table 2). MKL is our method with magnitude enhancement only, and NTCE-KD is with both magnitude and diversity enhancements of the non-target class.

Overall, both our methods, MKL and NTCE-KD, consistently outperform all compared methods in all settings. In over half of the cases, such as ResNet-56/ResNet-20, vgg13/vgg8, and ResNet-32×4/ShuffleNetV2, the students even outperform the teachers. Specifically, NTCE-KD surpasses both logit-based and feature-based methods by a considerable margin. Without diversity enhancement, MKL also demonstrates favorable performance compared with logit-based methods and achieves comparable or even superior performance compared with feature-based methods.

**Table 1:** Results of CIFAR-100 validation. Teachers and students are in the same architecture. MKL (magnitude-enhanced KL divergence): our method with magnitude enhancement only. NTCE-KD: our method with both magnitude and diversity enhancements. The best and second-best results are emphasized in **bold** and underlined cases.

Method	Teacher	ResNet-56 72.34	ResNet-110 74.31	ResNet-32×4 79.42	WRN-40-2 75.61	WRN-40-2 75.61	vgg13 74.64
	Student	ResNet-20 69.06	ResNet-32 71.14	ResNet-8×4 72.5	WRN-16-2 73.26	WRN-40-1 71.98	vgg8 70.36
Feature	FitNet (ICLR15)	69.21	71.06	73.5	73.58	72.24	71.02
	AT (ICLR17)	70.55	72.31	73.44	74.08	72.77	71.43
	RKD (CVPR19)	69.61	71.82	71.9	73.35	72.22	71.48
	OFD (ICCV19)	70.98	73.23	74.95	75.24	74.33	73.95
	CRD (ICLR20)	71.16	73.48	75.51	75.48	74.14	73.94
	ReviewKD (CVPR21)	71.89	73.89	75.63	76.12	75.09	74.84
	CAT (CVPR23)	71.62	73.62	76.91	75.60	74.82	74.65
Logits	KD (NIPS14)	70.66	73.08	73.33	74.92	73.54	72.98
	DKD (CVPR22)	71.97	74.11	76.32	76.24	74.81	74.68
	CTKD (AAAI23)	71.19	73.52	73.39	75.45	73.93	73.52
	DOT (ICCV23)	71.07	73.72	75.12	75.85	74.06	73.77
	LS (CVPR24)	71.43	74.17	76.62	76.11	74.37	74.36
	**MKL (ours)**	**72.16**	**74.41**	**76.91**	**76.58**	**74.92**	**74.89**
	**NTCE-KD (ours)**	**73.46**	**75.71**	**78.66**	**77.65**	**76.44**	**76.33**

**Table 2:** Results of CIFAR-100 validation. Teachers and students are in different architectures. MKL (magnitude-enhanced KL divergence): our method with magnitude enhancement only. NTCE-KD: our method with both magnitude and diversity enhancements. The best and second-best results are emphasized in **bold** and underlined cases.

Method	Teacher	ResNet-32×4 79.42	WRN-40-2 75.61	vgg13 74.64	ResNet-50 79.34	ResNet-32×4 79.42
	Student	ShuffleNetV1 70.5	ShuffleNetV1 70.5	MobileNetV2 64.6	MobileNetV2 64.6	ShuffleNetV2 71.82
Feature	FitNet (ICLR15)	73.59	73.73	64.14	63.16	73.54
	AT (ICLR17)	71.73	73.32	59.4	58.58	72.73
	RKD (CVPR19)	72.28	72.21	64.52	64.64	73.21
	OFD (ICCV19)	75.98	75.85	69.48	69.04	76.82
	CRD (ICLR20)	75.11	76.05	69.73	69.11	75.65
	ReviewKD (CVPR21)	77.45	77.14	70.37	69.89	77.78
	CAT (CVPR23)	78.26	77.35	69.13	71.36	78.41
Logits	KD (NIPS14)	74.07	74.83	67.37	67.35	74.45
	DKD (CVPR22)	76.45	76.70	69.71	70.35	77.07
	CTKD (AAAI23)	74.48	75.78	68.46	68.50	75.31
	DOT (ICCV23)	74.58	75.92	68.21	68.36	75.55
	LS (CVPR24)	75.62	76.62	68.61	69.02	75.56
	**MKL (ours)**	**76.81**	**77.01**	**70.13**	**70.52**	**77.10**
	**NTCE-KD (ours)**	**78.43**	**78.66**	**72.06**	**72.85**	**79.43**

ImageNet-1k. The experimental results of ImageNet-1k are presented in Table 3 in terms of Top 1 and Top 5. Our method, NTCE-KD, excels compared to other methods on the large-scale dataset. And MKL notably outperforms the majority of approaches in both Top 1 and Top 5 accuracy, achieving near-optimal performance. For identical architectures, MKL achieves state-of-the-art (SOTA) performance, surpassing feature-based methods by 0.46% and logit-based methods by 0.37%. For different architectures, MKL attains SOTA performance in Top 5 and secures a suboptimal position in Top 1.

### 4.3. Ablation Study

We conduct ablation studies in terms of magnitude and diversity enhancements, and the results are shown in Table 4. Considering ① and ②, the results show that magnitude enhancement benefits the student models, with improvements of 1.36%, 1.66%, and 3.58%. Similarly, the diversity enhancement benefits the student models with improvements of 1.9%, 1.82%, and 3.13%. Considering ① and ④, magnitude and diversity enhancements have orthogonal effects on model improvement, and their combination can further enhance the student models.

### 4.4. Analysis of Magnitude Enhancement

**Figure 4:**
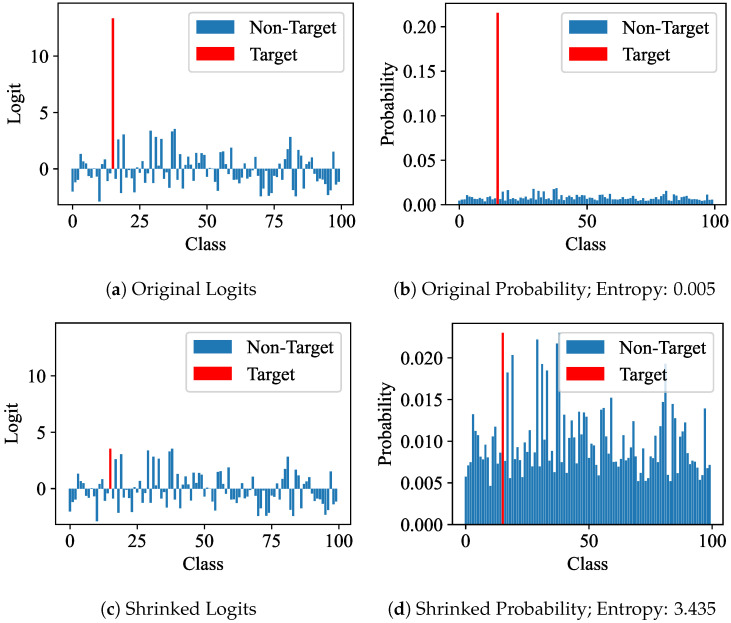
Comparison between logits and probabilities before and after magnitude enhancement.

Logits and probabilities. We visualize the logits and probabilities before and after multi-shrinking the target class of the logits. We observe that the target class of logits is more prominent than the non-target class, as illustrated in Figure 4a. And the target class of probabilities becomes excessively prominent after softmax, as depicted in Figure 4b, leading to an insufficient optimization of the non-target class. Our approach, MKL, shrinks the target class of the logits, resulting in balanced target and non-target classes of the multi-shrunk logits and probabilities, as shown in Figure 4c,d. Moreover, with the enhancement in magnitude, the entropy of probabilities undergoes a significant increase from 0.005 to 3.435, effectively resulting in richer soft labels, as shown in Figure 4b,d.

**Figure 5:**
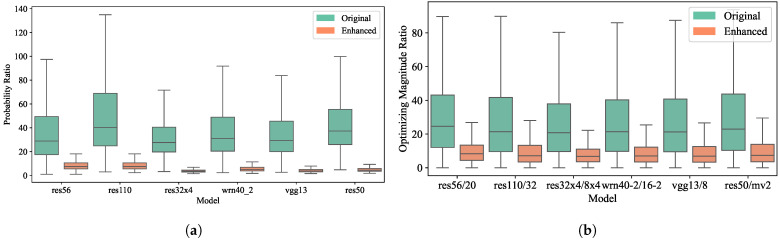
Comparison of probability ratio and optimizing magnitude ratio before and after magnitude enhancement. (**a**) r1: ratio of the probability of the target class to that of the non-target classes; (**b**) r2: ratio of the optimizing magnitude of the target class to that of the non-target classes.

Target/non-target ratio. For a more thorough analysis of the target and non-target classes within soft labels, we compare the probability ratio r1 and the optimizing magnitude ratio r2 (in Equation (11)) between the target class and the average non-target class before and after augmentation on the CIFAR-100 dataset. As shown in Figure 5, we find that the probability ratio is around 40 across all teacher models, with some even exceeding 100, and the optimizing magnitude ratio is over 20. After magnitude enhancement, both ratios decreased to acceptable single-digit values, allowing for a more equitable optimization of the non-target class compared to the target class.

**Figure 6:**
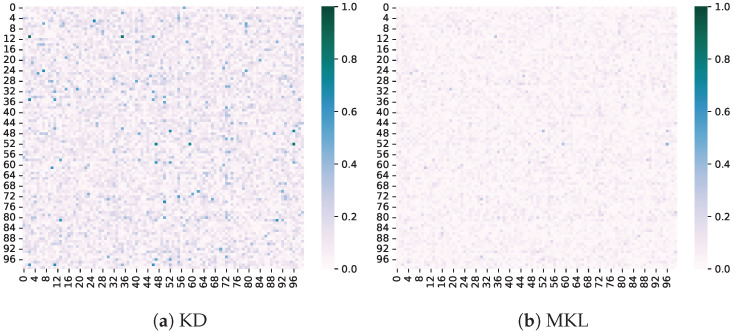
Difference between the teacher and student models of non-target classes.

Difference in non-target classes. We also compare the difference in non-target class logits between the teacher and student models. It can be observed that enhancing the values of non-target classes can prioritize their optimization, as shown in Figure 6, further aligning the outputs between the teacher and student models. This validates the significant role of the non-target class in knowledge distillation.

### 4.5. Analysis of Diversity Enhancement

Effect of diversity. We empirically investigate the impact of the conventional data augmentation strategy (DA) and the diversity-based data augmentation strategy (DDA) on various knowledge distillation methods, as illustrated in Figure 5. Here, $\Delta_{1}$ and $\Delta_{2}$ represent the performance improvements attained by DA and DDA, respectively. Overall, both data augmentation strategies demonstrate performance enhancements for all knowledge distillation methods ($\Delta_{1},\Delta_{2}>0$). However, compared to DA, our diversity-based data augmentation exhibits a noticeable performance gap ($\Delta_{2}>\Delta_{1}$) because DDA prioritizes enhancing the diversity of the non-target class, thereby endowing the augmented samples with richer knowledge during distillation.

Compatibility with SOTA methods. We explore the compatibility of DDA with other SOTA methods, as illustrated in Table 5. Our DDA consistently enhances both logit-based and feature-based knowledge distillation methods. DDA is specifically designed to enhance the diversity of non-target classes, and experimental results demonstrate its positive impact on other distillation methods, further underscoring the importance of the non-target class in knowledge distillation.

### 4.6. Determination and Sensitivity of Hyper-Parameters

Loss weights. α and β play a crucial role in balancing the contributions of the cross-entropy loss $L_{CE}$ and the distillation loss $L_{KD}$ to the overall optimization objective. α is set to 1.0, following most works [15,16,21,34,36]. β is determined through a grid search by selecting the value with the highest accuracy among different values: {“1.0”, “2.0”, “4.0”, “8.0”, “10.0”}, as shown in Table 6. Ultimately, we opt for β=8.0 as the distillation loss weight, as it yields the highest Top 1 accuracy in our experimental evaluations.

Based on the experimental results presented in Table 6, β initially has a significant impact on the model’s performance, peaking at β=8.0. However, after β exceeds 4.0, the improvement in accuracy becomes marginal, indicating that the model is less sensitive to further increases in β. This underscores the robustness within a certain range and the importance of the distillation loss in effectively transferring knowledge from the teacher to the student model. Conversely, the accuracy in Table 7 remains relatively stable across varying α values, indicating a lower sensitivity to this parameter. The sensitivity analysis reveals that optimizing β is crucial for maximizing knowledge distillation effectiveness, as the distillation loss plays a pivotal role in capturing the knowledge transfer.

Shrinkage coefficients. $D_{\lambda }$ is set to [1,0.5,0] for the best performance in our experiments. This choice of $D_{\lambda }$ indicates that our method is relatively robust to variations in the shrinkage coefficients, as the accuracies remain close across different settings in Table 8. However, the small improvement in accuracy when using [1,0.5,0] compared to other configurations suggests that the selection of these coefficients could have a non-negligible impact on the overall performance. Therefore, further exploration of optimal shrinkage coefficients for different datasets and model architectures remains an interesting direction for future work.

### 4.7. Analysis of Computational Complexity

In this section, we analyze the computational complexity of our proposed method, particularly considering the addition of non-target class optimization. During the loss computation phase, our approach necessitates the computation of probabilities and KL divergence for each shrinkage coefficient. This leads to a computational complexity of O(kn), where *k* represents the number of shrinkage coefficients.

Notably, when *k* is equal to 1, our approach does not introduce any additional overhead. Moreover, the predominant cost during training stems from the forward passes of the teacher and student models. Our enhancement lies primarily in the loss computation, which, compared to the forward passes, can be considered negligible. Additionally, our method does not introduce any additional overhead during the testing phase. As demonstrated in Table 9, the training time of our method is comparable to other logit-based methods, yet it achieves significantly higher accuracy.

## 5. Discussion

The NTCE-KD approach, primarily evaluated in the context of image classification, holds significant promise for broader applications beyond this domain. Specifically, its principles can be extended to tasks such as person re-identification (ReID) [37,38,39,40,41] and 3D point cloud understanding [42,43,44].

In the realm of person ReID, which involves identifying individuals across multiple camera views, challenges like occlusions, pose variations, and clothing changes are prevalent. NTCE-KD can address these challenges effectively by emphasizing non-target classes. By augmenting gradients of non-target classes, the model can learn features that discriminate better between similar individuals, leading to more robust representations.

Similarly, in 3D point cloud understanding tasks, such as object classification, segmentation, and detection, distinguishing between target and non-target classes is fundamental. NTCE-KD can enhance the model’s ability to discern subtle differences between similar objects within point clouds. By focusing on non-target class gradients, the model learns features that generalize well across instances.

Although the current research primarily focuses on validating NTCE-KD in image classification, its potential for person ReID and 3D point cloud understanding tasks is promising. Initial experiments in Appendix A suggest that NTCE-KD effectively leverages non-target class information to improve model performance in these domains. However, further investigation and experimentation are necessary to fully explore its capabilities.

In conclusion, the NTCE-KD approach offers a versatile framework applicable to a broader range of tasks beyond image classification. Future research will delve into its effectiveness in person ReID and 3D point cloud understanding, with the aim of conducting comprehensive experiments to validate its efficacy in these domains.

## 6. Conclusions

In this paper, we propose a novel knowledge distillation method, termed NTCE-KD, which enhances the non-target class from both magnitude and diversity perspectives to improve the distillation process. The NTCE-KD method exhibits significant performance improvements on the CIFAR-100 and ImageNet-1k datasets. Furthermore, through extensive analytical experiments, we validate the effectiveness of our approach. While promising, our method’s reliance on a single-teacher model could limit its robustness. To address this, future work could explore multi-teacher knowledge distillation, which could provide richer knowledge to further enhance both performance and generalization. We believe this work contributes to the optimization of soft labels and logit-based distillation methods.

## Figures and Tables

**Table 3:** Results of ImageNet-1k validation. MKL (magnitude-enhanced KL divergence): our method with magnitude enhancement only. NTCE-KD: our method with both magnitude and diversity enhancements. The best and second-best results are emphasized in **bold** and underlined cases.

Method	Teacher/Student	ResNet34/ResNet18		ResNet50/MN-V1	
		Top 1	Top 5	Top 1	Top 5
	Teacher	73.31	91.42	76.16	92.86
	Student	69.75	89.07	68.87	88.76
Feature	AT(ICLR17)	70.69	90.01	69.56	89.33
	OFD(ICCV19)	70.81	89.98	71.25	90.34
	CRD(ICLR20)	71.17	90.13	71.37	90.41
	ReviewKD(CVPR21)	71.61	90.51	72.56	91.00
	CAT(CVPR23)	71.26	90.45	72.24	**91.13**
Logits	KD(NIPS14)	70.66	89.88	68.58	88.98
	DKD(CVPR22)	71.70	90.41	72.05	91.05
	LS(CVPR24)	71.42	90.29	72.18	90.80
	**MKL (ours)**	**72.07**	**90.69**	**72.79**	91.10
	**NTCE-KD (ours)**	**73.12**	**91.37**	**74.90**	**92.33**

**Table 4:** Results of ablation study. The experiments are conducted on CIFAR-100, with three teacher–student pairs. MKL: magnitude-enhanced KL divergence. DDA: diversity-based data augmentation. ✓ and × present whether the method was adopted or not, respectively.

#	Ablation	MKL	DDA	ResNet-56/20	WRN-40-2/16-2	ResNet-32×4/8×4
①	Baseline	×	×	70.66	74.92	73.33
②	Magnitude	✓	×	72.02	76.58	76.91
③	Diversity	×	✓	72.56	76.74	76.46
④	NTCE-KD	✓	✓	73.28	77.65	78.04

**Table 5:** Results of compatibility with SOTA methods. The experiments are conducted on CIFAR-100, with Resnet32×4 as teacher and Resnet8×4 as student. Ori: original knowledge distillation method. Ori + DA: original method with conventional data augmentation. Ori + DDA: original method with diversity-based data augmentation.

Method	Ori	Ori + DA	$\Delta_{1}$	Ori + DDA	$\Delta_{2}$
KD	73.33	74.96	1.63	75.83	2.50
ReviewKD	75.63	76.89	1.26	77.32	1.69
DKD	76.32	77.02	0.70	77.87	1.55
LS	76.62	77.63	1.01	78.20	1.58
MKL (ours)	76.91	77.95	1.04	78.66	1.75

**Table 6:** Results on different β. The experiments are conducted on CIFAR-100, with Resnet32×4 as teacher and Resnet8×4 as student. α is set to 1.0.

β	1.0	2.0	4.0	6.0	8.0	10.0
Acc	75.22	75.65	76.41	76.76	76.91	76.89

**Table 7:** Results of different α. The experiments are conducted on CIFAR-100, with Resnet32×4 as teacher and Resnet8×4 as student. β is set to 8.0.

α	0.2	0.5	1.0	2.0	4.0
Acc	76.40	76.71	76.91	76.83	76.78

**Table 8:** Results different α. The experiments are conducted on CIFAR-100, with Resnet32×4 as teacher and Resnet8×4 as student. β is set to 8.0.

$D_{\lambda }$	[0.5]	[1]	[1, 0]	[1, 0.5, 0]	[1, 0.67, 0.33, 0]
Acc	76.66	76.58	76.83	76.91	76.85

**Table 9:** Training time and accuracy of different methods. The experiments are conducted on CIFAR-100, with Resnet32×4 as teacher and Resnet8×4 as student.

Method	Feature			Logit		
	ReviewKD	OFD	CRD	KD	DKD	NTCE-KD
Time (ms)	25.84	19.54	41.03	10.51	11.74	11.89
Acc	75.63	74.95	75.51	73.33	76.32	76.91

## Data Availability

The two datasets (CIFAR-100 and ImageNet-1k) used to illustrate and evaluate the proposed method are publicly available.

## Abbreviations

The following abbreviations are used in this manuscript:

NTCE-KD	Non-Target-Class-Enhanced Knowledge Distillation
KD	Knowledge distillation
KL	Kullback–Leibler
MKL	Magnitude-enhanced Kullback–Leibler divergence
DDA	Diversity-based data augmentation strategy

## Appendix A. Results of Discussion

### Appendix A.1. Results of Person ReID

To investigate the applicability of our method, NTCE-KD, to person ReID, we conducted experiments following the experimental settings in [45]. We set ResNet34 and ResNet18 as the teacher and student models, respectively. We compared the performance of the student model trained with both the original KL divergence and our proposed magnitude-enhanced KL (MKL) divergence, as shown in Table A1. The results indicate a significant improvement in model performance through distillation. Furthermore, MKL outperforms KL, demonstrating that NTCE-KD is equally applicable to person reID.

**Table A1:** Results of Market1501 for person ReID. MKL: trained with our proposed magnitude-enhanced KL divergence. KL: trained with original KL divergence.

Method	Teacher	Student	KL	MKL
mAP	80.9	76.6	81.5	82.4

### Appendix A.2. Results of Point Cloud Analysis

To explore the effectiveness of our method, NTCE-KD, in point cloud analysis, we conducted experiments following the experimental settings in [46]. We set PointMLP and PointMLPElite as the teacher and student models, respectively. We compared the performance of the student model trained with both the original KL divergence and our proposed MKL divergence, as shown in Table A2. The results demonstrate a significant improvement in model performance through distillation. Moreover, MKL outperforms KL, confirming that NTCE-KD is equally applicable to point cloud classification.

**Table A2:** Results of ModelNet40 for person ReID. MKL: trained with our proposed magnitude-enhanced KL divergence. KL: trained with original KL divergence.

Method	Teacher	Student	KL	MKL
Acc	92.9	80.9	81.2	82.9
